# Current crisis or artifact of surveillance: insights into rebound chlamydia rates from dynamic modelling

**DOI:** 10.1186/1471-2334-10-70

**Published:** 2010-03-16

**Authors:** David M Vickers, Nathaniel D Osgood

**Affiliations:** 1 Interdisciplinary Studies, College of Graduate Studies and Research, University of Saskatchewan, Saskatoon, Saskatchewan, Canada; 2 Computer Science, College of Arts and Science, University of Saskatchewan, Saskatoon, Saskatchewan, S7N 5C9, Canada; 3 School of Public Health, University of Saskatchewan Saskatoon, Saskatchewan, Canada

## Abstract

**Background:**

After initially falling in the face of intensified control efforts, reported rates of sexually transmitted chlamydia in many developed countries are rising. Recent hypotheses for this phenomenon have broadly focused on improved case finding or an increase in the prevalence. Because of many complex interactions behind the spread of infectious diseases, dynamic models of infection transmission are an effective means to guide learning, and assess quantitative conjectures of epidemiological processes. The objective of this paper is to bring a unique and robust perspective to observed chlamydial patterns through analyzing surveillance data with mathematical models of infection transmission.

**Methods:**

This study integrated 25-year testing volume data from the Canadian province of Saskatchewan with one susceptible-infected-treated-susceptible and three susceptible-infected-treated-removed compartmental models. Calibration of model parameters to fit observed 25-year case notification data, after being combined with testing records, placed constraints on model behaviour and allowed for an approximation of chlamydia prevalence to be estimated. Model predictions were compared to observed case notification trends, and extensive sensitivity analyses were performed to confirm the robustness of model results.

**Results:**

Model predictions accurately mirrored historic chlamydial trends including an observed rebound in the mid 1990s. For all models examined, the results repeatedly highlighted that increased testing volumes, rather than changes in the sensitivity and specificity of testing technologies, sexual behaviour, or truncated immunological responses brought about by treatment can, explain the increase in observed chlamydia case notifications.

**Conclusions:**

Our results highlight the significant impact testing volume can have on observed incidence rates, and that simple explanations for these observed increases appear to have been dismissed in favor of changes to the underlying prevalence. These simple methods not only demonstrate geographic portability, but the results reassure the public health effort towards monitoring and controlling chlamydia.

## Background

With millions of new cases occurring annually, *Chlamydia trachomatis *is the most common cause of bacterial sexually transmitted infection (STI) worldwide [1]. Among women, the magnitude of morbidity associated with sexually transmitted chlamydia can be staggering [1,2]. Chronic and progressive disease due to unresolved chlamydia infections include endometritis, salpingitis, pelvic inflammatory disease, ectopic pregnancy [3-5], and has also been associated with an increased risk of human immunodeficiency virus infection and cervical dysplasia [1]. Given the detrimental impact that chlamydia infections can have on reproduction, currently observed rates, and how best to reduce them, have been at the forefront of national policy agendas in North America, Europe, and Australia [5-7].

Collectively, two noticeable epidemiological profiles among reported case rates have emerged throughout many developed countries [3,4]. The first of these profiles has been observed in Canada, Finland, Norway, and Sweden where, after initially falling in the face of intensified control efforts, reported rates of chlamydia infections have rebounded [3,4]. The second profile has been observed in the U.S., the U.K., and Australia where incidence rates have been steadily increasing throughout their entire reporting history [3]. Recent hypotheses for these rising trends of chlamydia have focused on the introduction of improved testing technologies, antimicrobial resistance, wide spread changes in the riskiness of sexual practices, and arrested immunity [4] (see Table 1). While the evidence supporting these hypotheses has been the subject of recent debate [4,8,9], their validity remains to be a focal point of current chlamydia research [9].

**Table 1:** Seven hypotheses for increasing Chlamydia rates modified from "Epidemiology of Chlamydia infection: are we losing ground? Rekart ML, Brunham RC, 84; 87-91, copyright 2008" [4] with permission from BMJ Publishing Group, Ltd.

H1.	More false positive tests because NAAT* methods have lower specificity than culture methods.
H2.	NAAT testing results in increased case detection due to better sensitivity than non-NAAT methods.
H3.	NAAT testing of urine is less invasive and more acceptable, particularly among men, resulting in higher testing rates.
H4.	NAAT methods allow for self-collected specimens and targeted screening among persons at high risk.
H5.	Decreased chlamydial susceptibility to antimicrobials.
H6.	Increased rates of unsafe sexual practices.
H7.	Arrested immunity resulting from treatment with antimicrobials.

* NAAT = Nucleic Acid Amplification Test.

None of the hypotheses in Table 1 are mutually exclusive, and several are likely operating simultaneously [4]. Therefore, understanding how these hypotheses are contributing to the dynamics of chlamydia will benefit from (but not entirely depend upon) techniques for modelling dynamic complexity. The primary advantage to using dynamical models is that they depict explicit statements about system structure and how the elements within the system interact [10]. As a result, they enable valuable insights into how certain behaviour has arisen over time [10,11]. We believe that this methodological strength is particularly well suited for elucidating the main drivers behind rebounding case counts of chlamydia infections. In this article, we discuss: one, the methodological approach we used to construct simple mathematical models of chlamydia transmission; two, how we were able to integrate them with observed data; and three, how we used this approach to, in the context of recent rebound hypotheses (Table 1), parsimoniously explain the current epidemiological profile of chlamydia infections in the Canadian province of Saskatchewan.

## Methods

### Data Sources and Trends

Saskatchewan has a population of approximately 1,014,649 people [12]. Of this number, approximately 7.7 per cent of its residents are between the ages of 15 and 19 years, 7.5% are between the ages of 20 and 24 years, and 84.8 per cent are ≥ 25 years of age. In Saskatchewan, 13 health regions collect surveillance data on reportable diseases, which are then reported to the Communicable Disease Division of the Saskatchewan Ministry of Health. Of the three-abovementioned age groups, those aged between 15 and 24 years comprise 64 to 76 per cent of all reported chlamydia cases in Saskatchewan [5].

Since 1984, chlamydia infections have been a reportable infectious disease in Saskatchewan. During this time, all reported cases of chlamydia have either been diagnosed based on clinical criteria (i.e., urethral discharge, burning on urination, irritation in the distal urethra, dysuria, abnormal vaginal discharge or menstrual bleeding, post-coital bleeding, and lower abdominal pain) [4,5], laboratory methods (i.e., culture, enzyme immunoassay or polymerase chain reaction), or both depending on the year.

Key, aggregated longitudinal data between 1983 and 2007 were assembled from a combination of Provincial Health Reports, records of the Provincial Laboratory, as well as the Public Health Agency of Canada. These data consisted of reported chlamydia case counts, incidence rates (per 100,000 population per year), and testing volume. Case notifications and incidence data are publically available, and was obtained through a combination of reviewing Public Health Reports of the Saskatchewan Ministry of Health, and from the Public Health Agency of Canada's Notifiable Diseases Online website where data gaps existed for historic years in Provincial records. Data of testing volumes is also publically available, and was provided to us by the Saskatchewan Ministry of Health upon request. No formal permission was required to use these data.

Between 1983 and 1991, chlamydia test volume data was combined with another category of viral testing. However, we were able to obtain documented viral testing levels for several years before 1983 (i.e., 1979-1982) from public health reports of the Saskatchewan Ministry of Health. Using the earliest data that separated chlamydia testing volumes (1991-1992) in conjunction with records of "combined" viral testing volumes (1982-1990) we were able to impute testing volumes for chlamydia, between 1983 and 1990. The "combined" category imposed an upper bound on the level of chlamydia testing that could have occurred. Each of the different imputation strategies was bounded within a narrow defined range of possible values that converged by 1990 (not shown). Because of this asymptotic consistency, the resultant imputed test volume data series did not affect the overall interpretation of the results we report below. From reported chlamydia case counts and testing volume, we were also able to derive a time series for prevalent chlamydia infection among those tested.

Two characteristics of this data gave us a unique advantage over previous studies on chlamydia transmission: the first was a 25-year reporting history of chlamydia. To our knowledge, there are few jurisdictions, worldwide, that have access to similarly broad data; the second was that all testing in the province has been done by one agency (i.e., the Provincial Disease Control Laboratory). This provided reliable testing volume (i.e., denominator) data over the entire reporting history.

**Figure 1:**
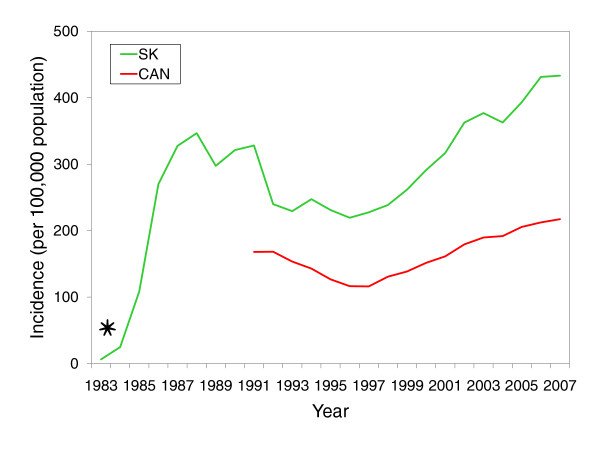
**Historic trends of *Chlamydia trachomatis *infections in Saskatchewan and Canada between 1983 and 2007**. Asterisk indicates when chlamydia infections became reportable in Saskatchewan (1984). Saskatchewan Incidence rates (per 100,000 population per year) are crude rates and are calculated as the ratio of number of reported cases and the population of Saskatchewan.

Some of the salient trends of reported chlamydia cases are displayed in Figure 1. This time series displays rapid growth from 1983 to the late 1980s - a factor that was undoubtedly fueled by the fact that chlamydia had become a reportable infection in 1984. This was followed by pronounced downward trend between 1988 and 1996. Since 1991, the province of Saskatchewan has recorded incidence rates up to two-times higher than national rates, and since 1997, an observable rebound has occurred.

### Model Structure and Formulation

The models we examined provided robust frameworks for data analysis. In particular, they were developed to integrate testing volume data, reproduce reported chlamydia case counts, while also seeking to understand the general epidemiological processes underlying these time series. Throughout this investigation, the above data contributed to adding confidence to model assumptions and structure. Model construction followed an iterative process where various causal hypotheses were translated into systems of differential equations. These equations were then simulated to determine whether they were capable of reproducing historical data using plausible parameter values derived from the chlamydia literature.

**Figure 2:**
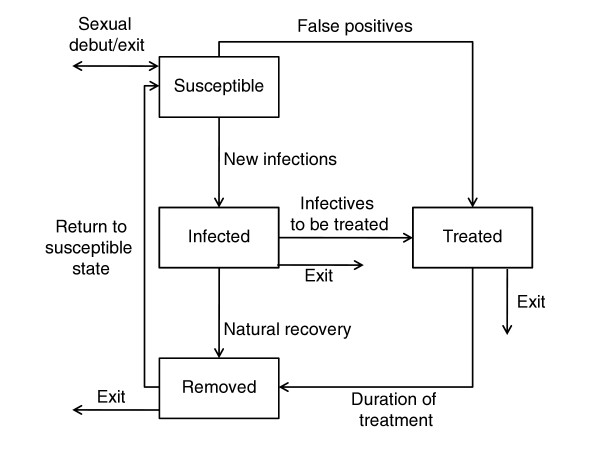
**Schematic stock and flow diagram of the susceptible-infected-treated-removed model**. We adopted a deterministic, compartmental, susceptible-infected-treated-removed-susceptible (or SITRS) framework. The susceptible class, *S*, contained those who were sexually active and could become infected; the infected class, *I*, contained those who were infectious; the treated class, *T*, containing both true and false positives; these people were assumed to have been tested, diagnosed as cases, and treated appropriately; the removed class, *R*, contained those who had naturally recovered from infection, and those who were given a positive diagnosis and temporarily reduced their sexual risk-taking behaviour after being treated. People in the removed class were assumed to eventually exit the sexually active population, or return to the susceptible class as a result of waned immunity or relapse into previous risky sexual behaviour. Additional model structures that were also investigated are displayed in Additional file 1.

Model structures were purposefully kept simple so to focus on broad insights into the processes that have shaped chlamydial patterns over time [13]. Through an extensive process of testing and evaluation (see below), several model structures were investigated. The model presented below emerged from that process as the most parsimonious dynamic hypothesis that adequately captured the historic patterns. However, we should note that the results we describe below remain consistent across the model structures we examined. Where possible, we try not to focus on the technical or mathematical details of model development. However, these details (including the other model structures examined) are outlined in the attached Additional file 1 or are available from the authors directly. An overview of the presented model structure is displayed in Figure 2.

We adopted a deterministic, compartmental, susceptible-infected-treated-removed-susceptible (or SITRS) framework [14,15]. The susceptible class, *S*, contained those who were sexually active and could become infected; the infected class, *I*, contained those who were infectious; the treated class, *T*, contained those who either sought health care or were found by contact tracing. Testing sensitivity and specificity were assumed to range between those of cell culture, enzyme immuno assays, and nucleic-acid amplification testing (NAAT). As a result, the treated class *T *contained both true and false positives; these people were assumed to have been tested, diagnosed as cases, and treated appropriately; these individuals were also assumed to abstain from risky sexual contact according to Public Health Agency of Canada guidelines [5] and remain "quarantined" in the treated class for the duration of treatment; the removed class, *R*, contained those who had naturally recovered from infection. This class also contained those who were given a positive diagnosis and temporarily reduced their sexual risk-taking behaviour after being treated. People in the removed class were assumed to eventually exit the sexually active population, or return to the susceptible class as a result of waned immunity or relapse into previous risky sexual behaviour (Figure 2).

The primary contribution of this approach is that it brings a unique perspective of integrating data with mathematical models in order to gain a novel understanding of chlamydial patterns that have been observed between 1983 and 2007. Model structure directly incorporated data for recorded test volumes as part of the testing assumptions explicitly captured by the model structure (please refer to "Model 1" in Additional file 1). Models were then calibrated to match case notification data. Integrating testing volume into the models placed a constraint on the model calibrations in order to match observed case counts. As a result of these data constraints, we were then able to "triangulate" an estimate of the prevalence of chlamydia infections in the population.

Other model structures examined the effect of treatment on the development of acquired immunity in combination with changes in sexual risk-taking behaviour (see Models 3 and 4 in Additional file 1). While acknowledging the importance of intricate biological and epidemiological differences by gender, age, and symptomotology our models followed the assumptions of Hethcote and Yorke [11] and ignored their effect. Instead, the models assume that chlamydia transmission occurs in one uniform homogeneous population of sexually active people. The population represented by these models therefore consists of those individuals at high risk, those who are efficient transmitters, as well as their sexual contacts.

### Parameter Values and Model Calibration

Initial parameter values were derived from available literature. Their values and ranges are summarized in Table 2. Model calibration, crosschecking, and sensitivity analyses were performed using a four-step process adapted from Van de Velde et al [16]:

**Table 2:** Baseline parameter values used during model calibrations.

Parameter	Description	Value (units)	References
	The mean number of susceptible individuals infected with chlamydia per year by an index case.	0.8-10 (1/year)	[27]
1/σ	Average duration of natural infection.	1.25* (years)	[27-30]
1/σ'	Average duration of infection when treated.	0.038* (years)	[5]
φ	Diagnostic test sensitivity.	0.50-0.92	[7]
φ'	Diagnostic test specificity.	0.98-1.0	[7]
1/μ	Average duration of sexual activity.	15^† ^(years)	[17,18]
1/δ	Average duration removed.	0.5-10^† ^(years)	[6,21]
*n*_*I*_	Number of infectives tested.	Calculated	
*n*_*S*_	Number of susceptibles tested.	Calculated	

*Fixed value based on references cited; † Assumption of parameter value based on references cited.

1. *Setting initial parameter values*: Each parameter value associated with the natural history of infection or treatment was estimated from key epidemiological or review articles in the available literature between 1997 and 2007. Where unavailable, parameter values (e.g., duration of sexual activity) were taken from modelling literature on gonorrhea infections [17,18]. Given that both gonorrhea and chlamydia are transmitted by similar behaviour, we think using data from other STIs is a reasonable assumption.

2. *Sampling parameter ranges and fitting the model*: Each parameter value was associated with a range of values found in the literature surveyed. Parameter settings that minimized the discrepancy between the historic reported case counts and those output by the model, were determined by a sequence of 50 optimizations using the Powell global optimization algorithm available in Vensim DSS for Windows (version 5.5c). This enabled different combinations of parameter vectors to be explored. Each optimization used a distinct random number seed, and performed approximately 1.0 × 10^6 ^simulations (totaling 5.0 × 10^7 ^simulations across all optimizations).

3. *Crosschecking model fit*: To build confidence in the model results, we compared model simulations to observed data series that were not used in step 2. These included the reported incidence rate (per 100,000 population per year) and fraction of positive cases among those tested.

4. *Sensitivity Analysis*: Each of the optimization scenarios identified a different point in parameter space that offered the "best fit" to the historic data. Because of this inherent variability present in each optimization scenario, we performed a sensitivity analysis based on the distribution of parameter vectors produced in step 2.

## Results

### Model Fit and Validation

**Figure 3:**
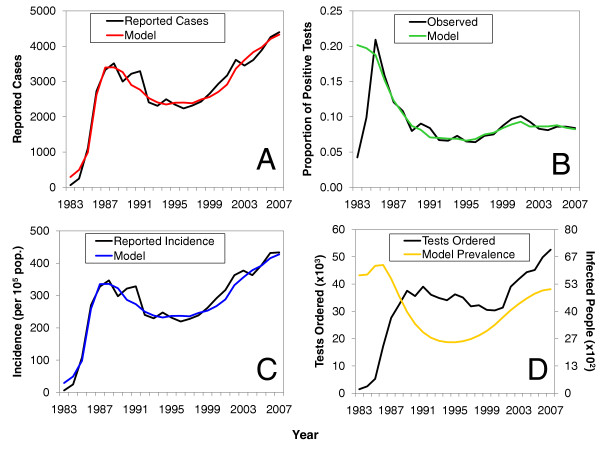
**Comparison of model calibrations to observed trends**. Calibrated numbers of (A) cases from the models compared to reported numbers of cases in Saskatchewan. Parts (B) and (C) cross-check the model to the observed proportion positive among those tested (B), and the reported incidence (per 100,000 population) (C). Model-generated curves in parts (A), (B), and (C) were arbitrarily chosen from 50 million optimization simulations. Part (D) is a visual comparison of testing volume between 1983 and 2007 to the "actual" prevalence in Saskatchewan generated by the model.

Figure 3 compares the observed historic trends in Saskatchewan and calibrated model simulations. In contrast to other seminal work on modelling STI transmission [11,17,18], a model that incorporates a "removed" state best reproduces observed chlamydia trends (see Figure 3 and Additional file 1, Figure S4). As shown, the model was able to accurately mirror observed temporal changes in reported case counts (Figure 3A). These trajectories also accurately reproduced the temporal changes in the observed incidence and the proportion positive among those tested without explicit instruction to match these data (Figures 3B and 3C, respectively).

One major advantage of this analysis was that it allowed us to produce a triangulated estimate of the "true" epidemiological state (i.e., the infected class *I*) that underlies the observed trends. Hereafter this will be referred to as the "actual" prevalence. As shown in Figure 3D, the model suggests that actual prevalence reached a maximum shortly after 1984, was in decline between 1991 and 1996, and was followed by an upward rebound between 1996 and 2003. It is interesting to note that while the model suggests that a rebound in the actual prevalence has indeed occurred, the peak of this upward trend lies below the peak attained in the mid 1980s. This behaviour suggests that the prevalence is moving towards an endemic steady state by a series of weakly damped oscillations - a familiar feature of the types of infectious disease models we studied here [14]. When the actual prevalence is superimposed on the observed trends in testing volumes, an obvious divergence between 2005 and 2007 is demonstrated (Figure 3D). Specifically, testing volume appears to be steadily increasing, while the actual prevalence has plateaued.

### Parameter Uncertainty

**Figure 4:**
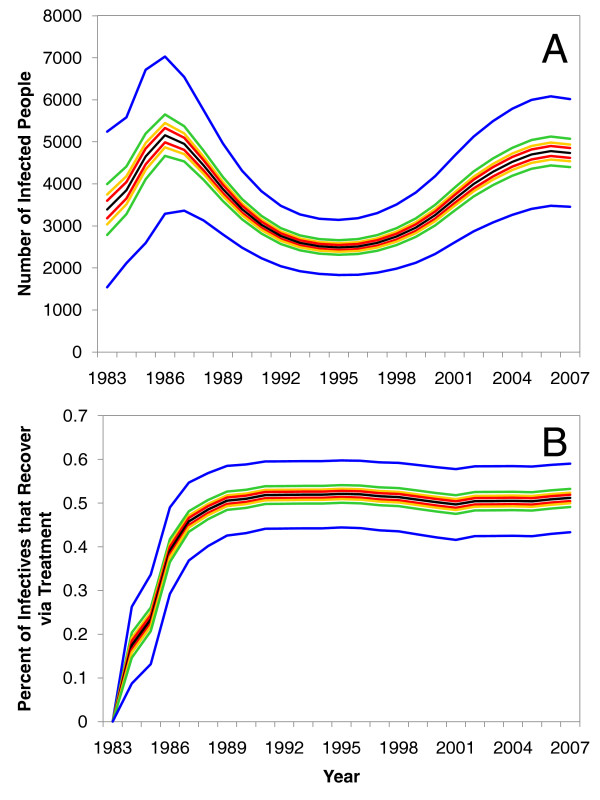
**Uncertainty and sensitivity analysis of model results**. Parts (A) and (B) are the results of a sensitivity analysis on the "actual" prevalence generated by the model and the fraction of infectives that have recovered via treatment, respectively. In both parts (A) and (B), the black line represents the mean value, and the coloured bands represent the 50 (red), 75 (yellow), 95 (green), and 99 per cent (blue) confidence intervals.

The parameter sets that best fit the observed epidemiological data produced a wide range of combinations. When we accounted for variability in the calibrated parameter values, the model's trajectories for the actual prevalence of chlamydia exhibited minor change and retained the same basic behaviour over time (Figure 4A). Even though this model did not explicitly simulate the efficiency of contact tracing, the model results suggest that the level of healthcare coverage (expressed as the fraction of recovering cases recovering through treatment) rapidly increased in the mid 1980s and has remained quite consistent over time (Figure 4B).

## Discussion

Globally increasing chlamydia rates have been widely discussed to be a result of both changes to testing technologies and changes in human sexual and social behaviour near the mid- to late-1990s [4,8,9]. Although initially intuitive, the results presented here provide evidence that such observed rebounding trends in chlamydia infections have resulted from a simpler set of oscillating epidemiological processes, in particular a significant delay in replenishing the susceptible population, that have been operating throughout the entire history of this infection. Taken together, our results demonstrate that currently observed rebounding chlamydia notifications are more likely a combined artifact of: one, when chlamydia infections became reportable; and two, the state of the underlying prevalence once surveillance was well-established (shortly after 1987), rather than because of fundamental changes that have arisen because of some of the hypotheses in Table 1 (see Additional file 1, Figure S5).

Several pieces of evidence suggest that adopting NAAT technologies have likely had an impact on observed chlamydia case counts. First, testing via NAAT methods is done on urine, which is more acceptable and easier to collect from high-risk youth and male patients [4]; this will, ultimately, allow more tests to be collected. Second, because of an increased sensitivity and decreased specificity profiles compared to non-NAAT methods, NAAT technologies allow for improved case detection [19-21]. While the improved sensitivity and decreased specificity profiles of NAAT methods are likely to impact observed chlamydia case rates [8], our models were able to reproduce observed trends without having to account for changes in test sensitivity or specificity since their introduction (which in many parts of Canada was between 2000 and 2001). Overall, our analysis is able to reinforce previous statements that higher testing frequency, alone, can have a significant influence on observed rates [8,21].

Additionally, our analysis suggests the existence of a positive feedback from testing volume that accounts for the recent continued climb in the reported chlamydia case counts between 2002 and 2006: a greater number of positive tests led to more awareness of the infection, which led to more testing being done, and still a larger number of positive tests (Figure 3D). However, higher rates of testing will also bring higher rates of treatment. Higher rates of treatment appear to have led to a reduction of the underlying prevalence over time, and thus contributed to the observed damped oscillations in the actual prevalence. Upon reflecting on the behaviour of the actual (model-derived) prevalence, we would also expect that observed rates will eventually begin to plateau as the prevalence among people tested begins to approach the actual prevalence in the population at risk.

The arrested immunity hypothesis posits that early treatment interrupts the immune response, thereby enhancing population susceptibility to infection as people re-enter the same sexual networks [4,22]. When we accounted for this phenomenon in two of the alternative model structures (Models 3 and 4 in Additional file 1), nearly identical results were observed when compared to a model that did not account for it (i.e., the model represented in Figure 2). The fact that the structures of Models 3 and 4 were able to accurately reproduce data, after being constrained by testing volume data, suggests that arrested immunity is very likely an important component of the underlying dynamics of chlamydia transmission. However, additional sensitivity analyses on Models 3 and 4 (not shown) revealed that the impact of arrested immunity at the population level may not be as significant as previously discussed [3]. Overall, this too seems to suggest that adverse immunological impacts of current test-and-treat polices are not required to explain the rebound.

Similarly, previous discussions in the literature have also implicated rebounding chlamydia rates to be a function of increased high-risk sexual behaviour that has, ultimately, resulted in an increase in both on-going and new chlamydia infections [4,6,9]. The models we presented here captured two different aspects of behaviour change. These were the mean number of susceptible individuals infected with chlamydia per year by an index case, , and the average duration a person stayed in the removed class, 1/δ. However, as with changes in testing technologies and arrested immunity, our models were able to reproduce observed trends without having to appeal to changes in sexual risk-taking behaviour.

There are some limitations to our analysis that need to be discussed. As with any model, the given structure represents a simplification of reality. We chose to develop a population-based model that is appropriate for exploring transmission dynamics in a large population where the infection is endemic. Therefore, we did not evaluate the impact of network structure or the duration of sexual partnership on our results. Although we may have described our results at the level of the individual, models of this type do not capture events that occur at the individual level. As a result, the models we present here will offer poor resolution for investigating network-based interventions. Despite this, these types of models are still capable of providing broad-level insights into historic and current epidemiology at the level of the population [23,24], and are able to shed light on specific questions in a way that alternative models, including human intuition and traditional epidemiology, do not.

Models are only as useful as the data available to inform them. Although our simple models contain many plausible relations that have some precedent in previous epidemiological observations, they were developed and calibrated in the absence of some important numerical data (e.g., data stratified by age and gender). Despite this simplification of reality, we do not think that stratifying the model by age or gender would have benefited the results presented here. This is largely because of inadequate provincial data to calibrate age and gender specific mixing parameters. In our opinion, calibrating a stratified model in the absence of such data would have suffered from overfitting. Regardless of these simplifying assumptions, one of the strengths of this analysis is that integrating surveillance data with dynamic models of chlamydia transmission allowed us to provide some underlying epidemiological context to data that has been largely criticized for lacking it [8,9]. More notably, these analyses have also highlighted how, once a surveillance system is well established (which in Saskatchewan was near 1987), and given a concerted effort to identify as many cases as possible, surveillance data (e.g., time series of testing volume) can mimic the underlying prevalence of infection (see Figure 3D and Additional file 1, Figure S5).

Although extant data limitations ruled out constructing a stratified model, two considerations give studying demographic heterogeneity priority for future work. First, having been able to explain the rebound in observed case notifications as a function of testing volume, collection of more detailed data could highlight contextual epidemiological differences among those being tested for chlamydia. Second, for these models to offer value in assessing intervention tradeoffs, it will be important to account for these population heterogeneities in the model structure. We are currently drawing on more-detailed data from a sub-provincial level (e.g., changes in contact rates, gender distribution amongst tested cases, the age distributions of contacts, rates of pelvic inflammatory disease, and changes in the social marketing of healthcare services over time) to expand the insights of the current analyses.

## Conclusions

With 25 years of data corresponding to over 804,000 tests and 69,000 cases this is likely one of the largest retrospective analyses on genital chlamydia. To help clarify the dynamic mechanisms underlying observed trends, the models presented here draw upon available indicators within existing data and integrate several cause-and-effect hypotheses. As a result, this has allowed us to critically examine the appropriateness of several key rebound hypotheses.

The primary aim of this study was to parsimoniously explain the recently observed rebound epidemiological profile of chlamydia since it became a reportable infection in the Canadian Province of Saskatchewan. By combining dynamic models with testing volume data to reproduce observed surveillance data, our results highlight the significant impact testing volume can have on observed case counts. The results of this study also illustrate the usefulness of our methods for deriving estimates of infection prevalence from freely available surveillance data. Overall, they provide a viable explanation for reported trends that appears to have been overlooked or dismissed in favor of hypotheses involving large-scale, aberrant changes to the underlying prevalence [21].

The calibrated models presented here offer value as tools for improving our understanding of chlamydia epidemiology. With some structural modification and additional data, they will be useful for examining how current trends might behave into the future under a variety of control scenarios. For example, they may be extended to explore the dynamics of introducing various candidate control policies, such as a nationally dedicated proactive screening program [6] or expedited partner therapy [25,26]. It bears emphasizing that while we have presented results from a single province with an exceptionally long record of chlamydia data, our methodological approach is both straightforward and general. Moreover, these methods offer geographic portability that can help inform the public health efforts in other contexts that collect similar information to that used here. Most importantly, these results reassure the public health effort towards monitoring and controlling chlamydia.

## Competing interests

The authors declare that they have no competing interests.

## Authors' contributions

Conceived models and designed experiments: DMV and NDO. Performed the simulation and calibration experiments: DMV and NDO. Analyzed and interpreted the results: DMV and NDO. Wrote the paper: DMV and NDO. All authors have read and approved the final manuscript.

## Pre-publication history

The pre-publication history for this paper can be accessed here:

http://www.biomedcentral.com/1471-2334/10/70/prepub

## Supplementary Material

Additional file 1 **Additional Model Structures and Supporting Information**. This file contains specifics of the mathematical structure, as well as some additional results of the models discussed in the main text. Click here for file
