# Supplementary material for: Current crisis or artifact of surveillance: insights into rebound chlamydia rates from dynamic modelling
Source: BMC Infect Dis. 2010 Mar 16;10:70. doi: 10.1186/1471-2334-10-70 (PMC2848153; doi:10.1186/1471-2334-10-70)
Supplement: Additional file 1 — Additional Model Structures and Supporting Information. This file contains specifics of the mathematical structure, as well as some additional results of the models discussed in the main text. [file 1471-2334-10-70-S1.DOC]

**Additional Files**

**Additional file 1 - Additional Model Structures and Supporting Information**

For the description of the transmission dynamics of an infectious disease, it has been traditionally convenient to divide the population into mutually exclusive classes, or compartments, with which numbers, or proportions, of the population “flow” through over time [11, 12-14]. The susceptible class, *S*, contains those who can become infected, the infectious class, *I*, and the removed class, *R*, who have acquired some degree of protective immunity. Much of the preliminary structural assumptions combine aspects of models previously published for studying gonorrhea [17] and chlamydia [22, 27], and were devoted to specifying the interaction of infection spread in the presence of readily-accessible healthcare provision.

A number of the assumptions in the model we presented in the main text have other plausible alternatives. We must therefore demonstrate the extent to which our main results and predictions are robust to different epidemiological assumptions and model structures. To do so, we investigated three alternative models that are described in more detail below. In every case we observed that: one, we can explain the observed data with relatively simple models; and two, all four of the models do not have to appeal to special factors to explain the recent rise in observed cases (i.e., changes in the sensitivity of diagnostic tests, sexual behaviour, or the arrested development of immunity because of treatment). We should note that the structure of “Model 2” (below) was our initial dynamic hypothesis (i.e., our true “Model 1”). However, for the sake of convenience (and hopefully not confusion) we refer to the model presented in the main text of the paper as “Model 1”.

**Model 1: A Simple Susceptible-Infected-Removed Model with Treatment of Infectives**

For this model, a compartmental *SITRS* (susceptible, infected, treated, removed, susceptible) framework was adopted [14-15]. The removed class *R*(*t*) was assumed to contain a combination of those who had not presented for treatment and recovered naturally from infection, as well as those who had temporarily changed their sexual risk taking behaviour. These were assumed to occur at the rates $T\sigma'$and $\sigma I$, respectively. Thus, Model 1 assumed that being found to have an infection, either symptomatic or asymptomatic, will have a temporary impact on risky sexual behaviour.

The fractions of the population that were susceptible, infected, treated, or were removed at time *t* were denoted by *S*(*t*), *I*(*t*), *T*(*t*), and *R*(*t*) respectively. We assumed that the sexually active population had a constant size *N,* where *N* = *S* + *I* + *T* + *R*. The “actual” or “true” prevalence of infection in the sexually-active population is represented by *I*(*t*). People were assumed to enter the susceptible state at sexual debut at a constant rate,$\mu N$, and exited at a rate$\mu S$. The probability of chlamydia transmission per year for a given partnership between a susceptible and infected individual is given by$\beta c$, hereafter denoted$\hat{\beta}$. Thus, for a given fraction of infected people in the population, , the number of susceptibles that become infected per year is . The number of false positives detected for a given test was assumed to occur at a rate $n_{S}\left(1-\phi'\right)()$. Here, the number of susceptibles tested is the difference between the recorded testing volume, *V*, and the number of infectives tested, $n_{S}=V-n_{I}$. The number of infectives tested was a function of the fraction of infectives tested, $\beta_{T}\left(1-e^{-\alpha\left(\frac{V}{I}\right)}\right)$, and the prevalence of infection in the population, *I*: $n_{I}=\beta_{T}\left(1-e^{-\alpha\left(\frac{V}{I}\right)}\right)I$. Our testing assumptions here posit a relation that states if testing increases, more cases will be found. However, simply doubling the number of tests does not mean that twice the number of infection will be found. In effect, the model assumes a “law of diminishing returns” where there is a lower and lower incremental benefit with increased testing. Thus, when testing volume, *V*, approaches zero, the fraction of infectives tested approaches zero at a rate $\alpha$; as testing volume increases, the fraction of infectives tested will approach$\beta_{T}$, reflecting that even with great testing effort, it takes a certain amount of time to identify some infectives. The fraction of infected people detected by testing (per year) were treated at a rate $n_{I}\phi$, recovered naturally at a rate$\sigma I$, or exited the infected class at a rate$\mu I$.

Those deemed infected (i.e., both true and false positives) were treated and returned to the susceptible class at a rate$T\sigma'$ or exited the treated class at a rate$\mu T$. While both seeking healthcare services and contact tracing are likely two separate processes responsible for bringing infected individuals to the treated state, we assumed that there is some *net time*, $n_{I}\phi$, that will result (that is mathematically equivalent to representing two separate rates). Keeping these as a single parameter helped reduce the risk of overfitting. For simplicity we made an assumption that those being treated follow the recommended clinical guidelines published by the Public Health Agency of Canada that patients abstain from risky sexual contact for 7-14 days whilst being treated. All those in the removed state then were assumed to return to the susceptible class as a result of waned immunity or relapse into previous risky sexual behaviour at a rate$\delta R$. Under these assumptions, the flow of individuals between the *S*, *I*, *T*, and *R* classes is depicted in Figure 2 (main text) and described by the following system of equations:

(1)

(2)

(3)

$\frac{dR}{dt}=I\sigma+T\sigma'-R\left(\delta+\mu\right)$. (4)

**Model 2: Chlamydia Transmission via a Susceptible-Infected-Treated Model**

For this model, a compartmental *SITS* (susceptible, infected, treated, susceptible) framework was adopted. This model provided a starting theoretical framework to use in drawing simple conclusions about the epidemiology of chlamydia. It made use of the notion that susceptibles become infectious, and then susceptible again. Because chlamydia is a reportable and curable infection, this model also assumed that a fraction of infectives were tested, treated, and then, too, become susceptible again. As with Model 1 (above), Model 2 made the assumptions that chlamydia infection occurs in one uniform homogeneous population, and that there are negligible periods of latency. In contrast to the other models examined, Model 2 made the assumption of negligible periods of protective immunity (i.e., no *R* state). The population represented by the model consisted of the segment of the sexually-active population who are at high-risk or are efficient transmitters (following the assumptions of Hethcote and Yorke [11]). This model also did not account for differences in sexual activity and it ignored epidemiological differences between men and women.

The movement between *S*, *I*, and *T* classes, based on the above assumptions, was described by the following system of ordinary differential equations (see Figure S1 for schematic diagram):

(5)

(6)

(7)

**Figure S1. Schematic stock and flow diagram of Model 2.**


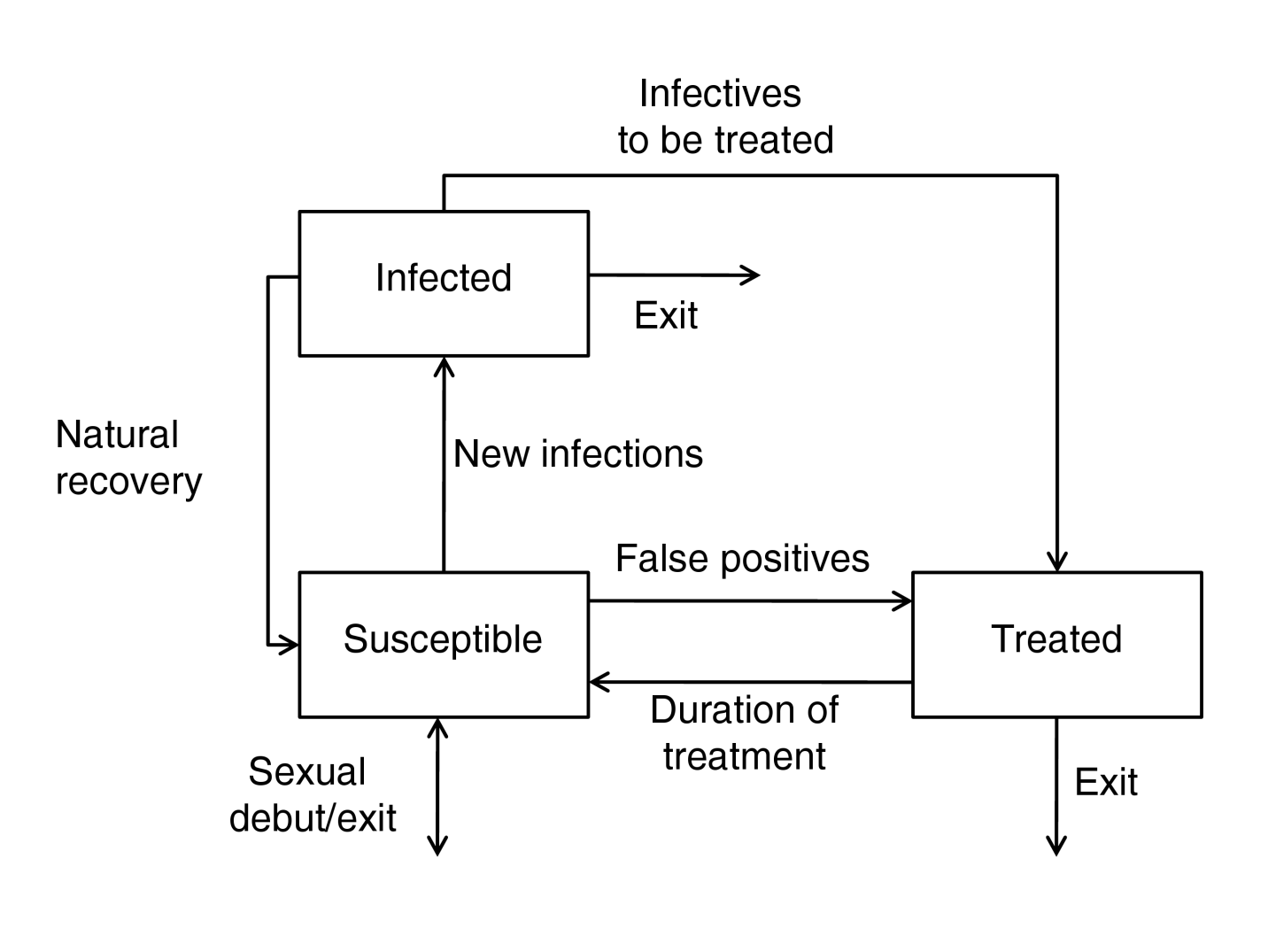


**Model 3: A Model that Assumes Treatment of Prevalent Infection Truncates Transient Acquired Immunity**

Here, we made an additional assumption adopted from Brunham et al., [22] that being found to have infection, and thus being treated, did not impact a person’s sexual risk-taking behaviour. In contrast to Model 1, our assumptions in Model 3 were motivated by the observation that being treated may arrest the development of acquired immunity, and thus returned a person to the susceptible state at a rate $T\sigma'$. As was mentioned in the main text, this assumption jointly accounted for no change in sexual risk behaviour, but also assumed that treatment arrested any benefit of transiently acquired immunity [4,22]. These additional assumptions produced the following system of equations (see Figure S2 for schematic diagram):

(8)

(9)

(10)

$\frac{dR}{dt}=I\sigma-R\left(\delta+\mu\right)$. (11)

**Figure S2. Schematic stock and flow diagram of Model 3.**


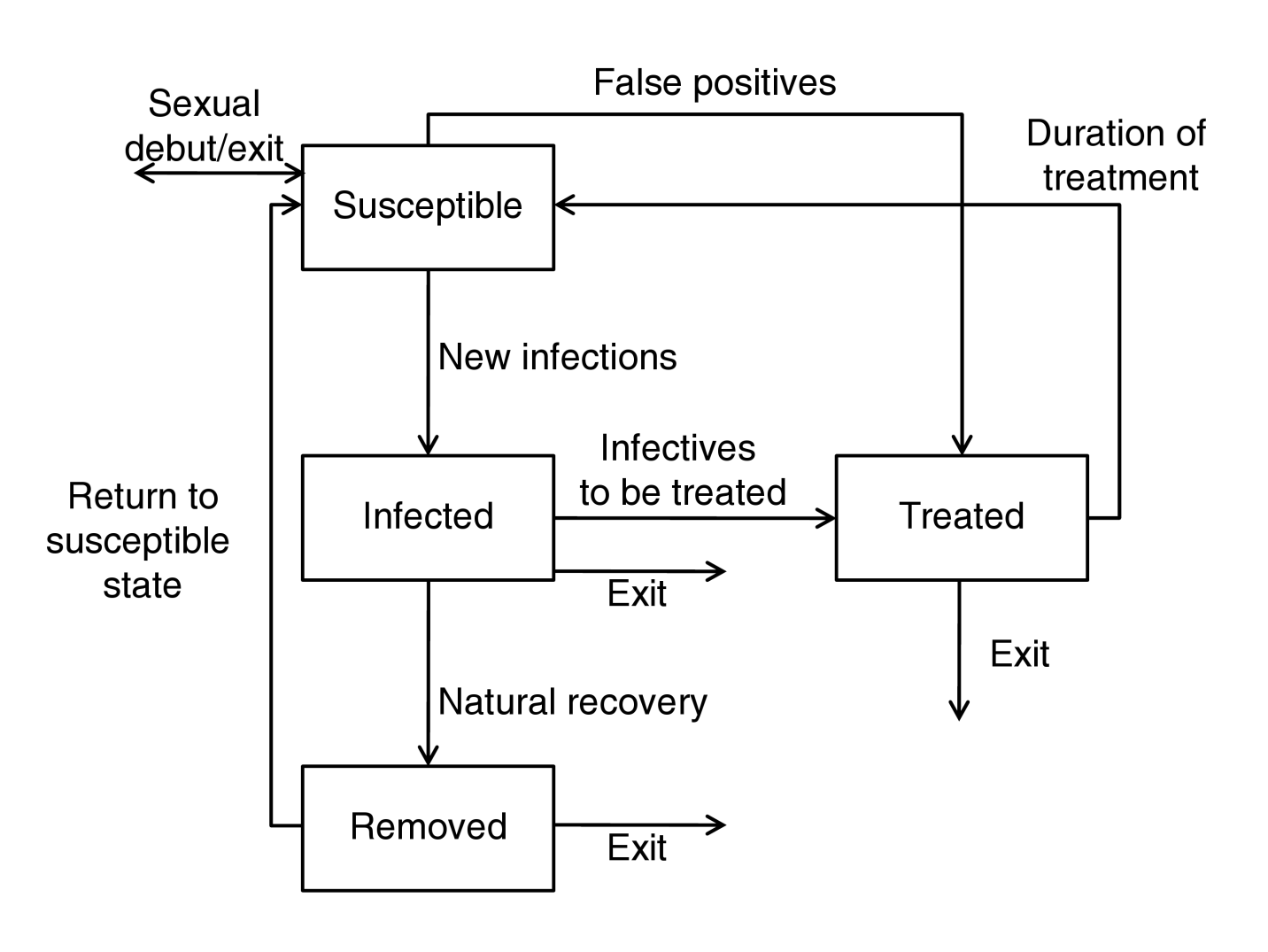


**Model 4: A Model that Combines Models 1 and 3**

Here, we combined our assumptions in Models 1 and 3. More specifically, the removed class *R*(*t*) was assumed to contain a combination of those who had not presented for treatment and recovered naturally from infection, and a fraction,$\theta$, of those who had temporarily changed their sexual risk taking behaviour after being treated. In addition to this, we also considered a scenario that jointly accounted for the lack of developing acquired immunity and no changes in sexual risk behaviour after being treated. This additional assumption returned the remaining fraction of the population, $\left(1-\theta\right)$, to the susceptible state at a rate $\left(1-\theta\right)T\sigma'$. These additional assumptions produced the following system of equations as well as the schematic diagram in Figure S3:

(12)

(13)

(14)

$\frac{dR}{dt}=I\sigma+\theta T\sigma'-R\left(\delta+\mu\right)$. (15)

**Figure S3. Schematic stock and flow diagram of Model 4.**


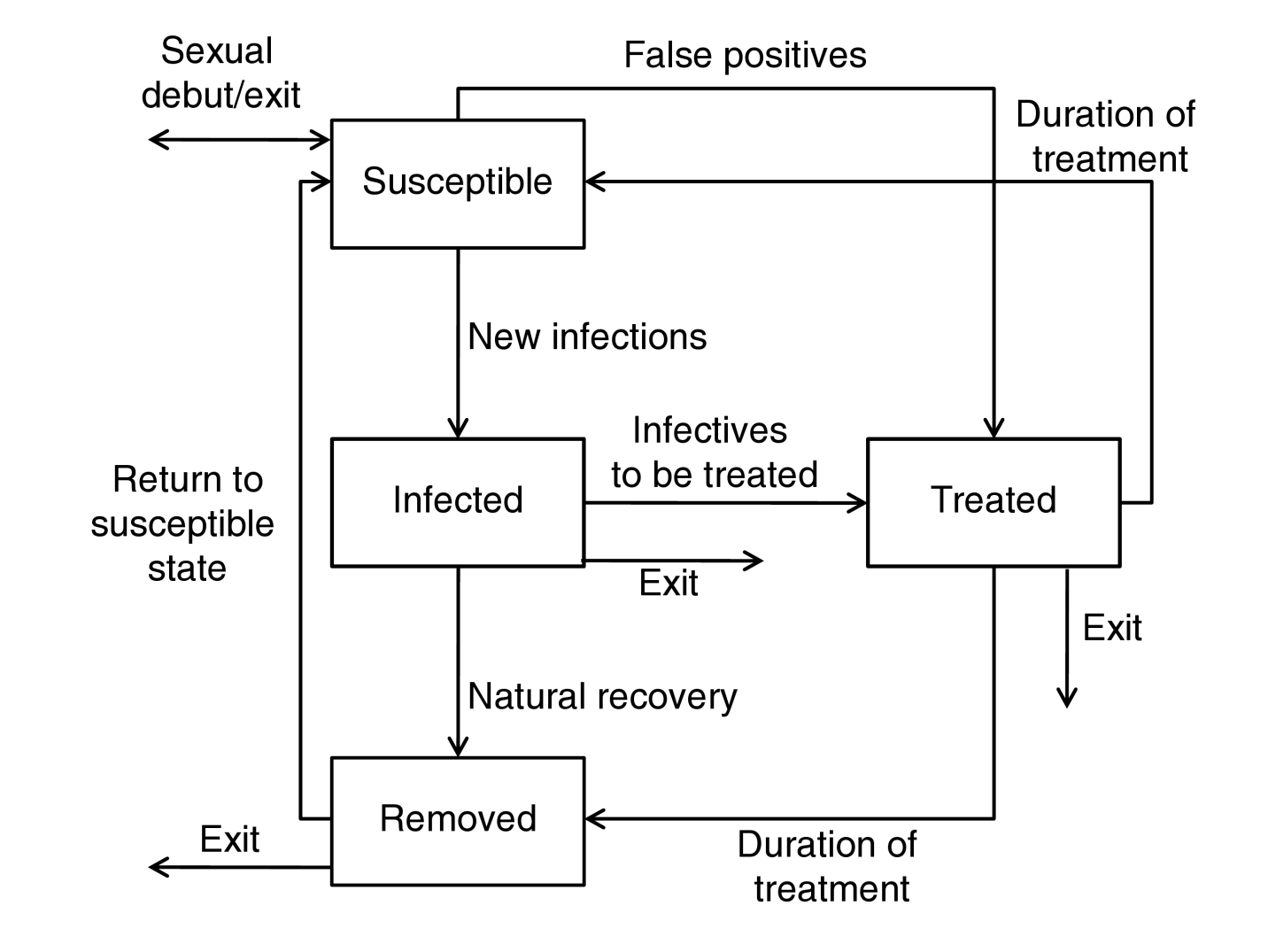


**Figure S4. Calibrated numbers of cases notifications from Models 2-4 compared to reported numbers of cases in Saskatchewan.**


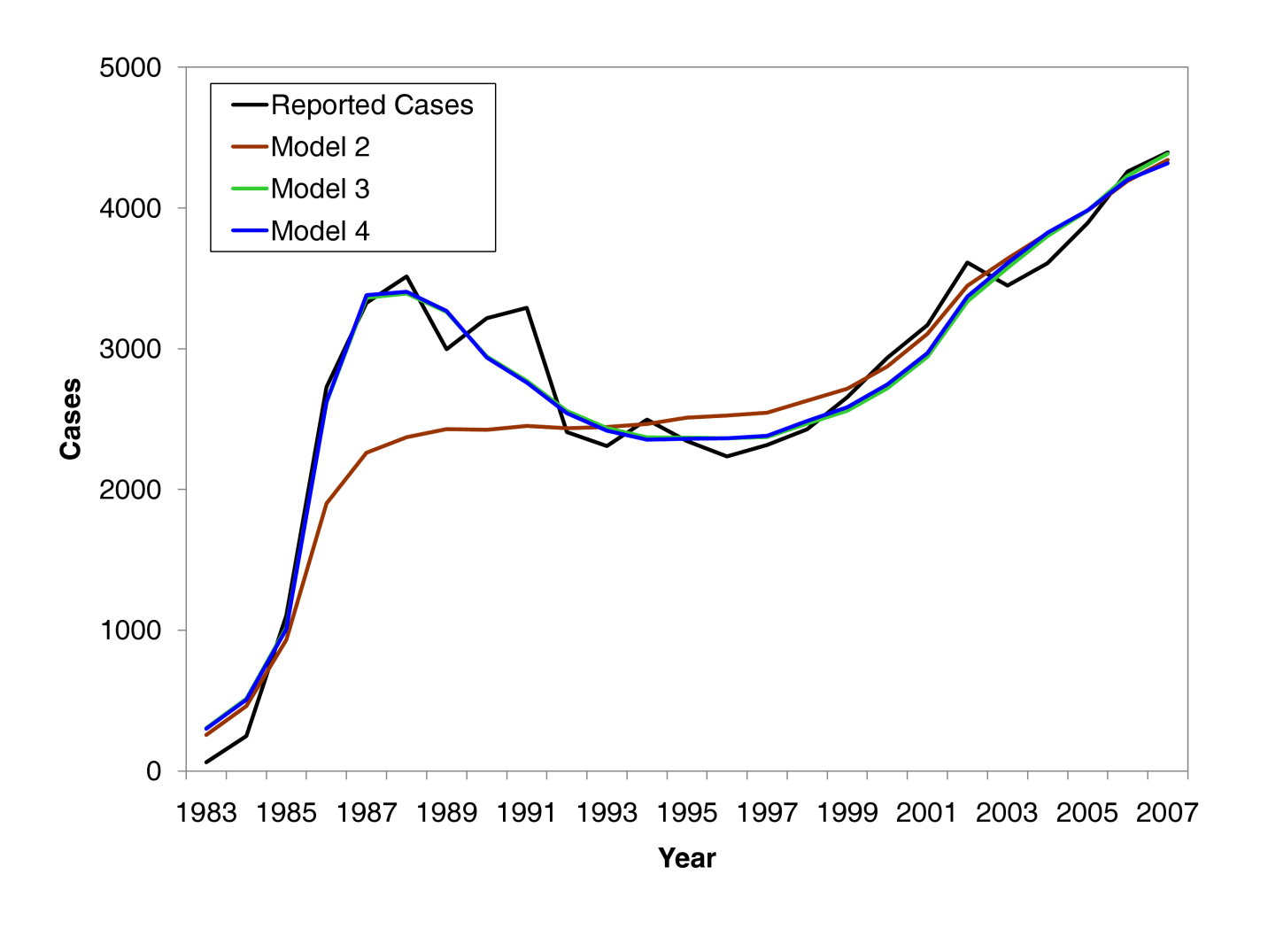


**Figure S5. A comparison of testing volume between 1983 and 2007 to the “actual” prevalence in Saskatchewan predicted by Models 2-4. Curves demonstrate that currently observed rebounding chlamydia notifications are likely a combined artifact of: one, when chlamydia infections became reportable; and two, the state of the underlying prevalence once surveillance was well-established (shortly after 1987). After 1987, the curves also illustrate how surveillance data can capture the underlying prevalence of infection.**

**
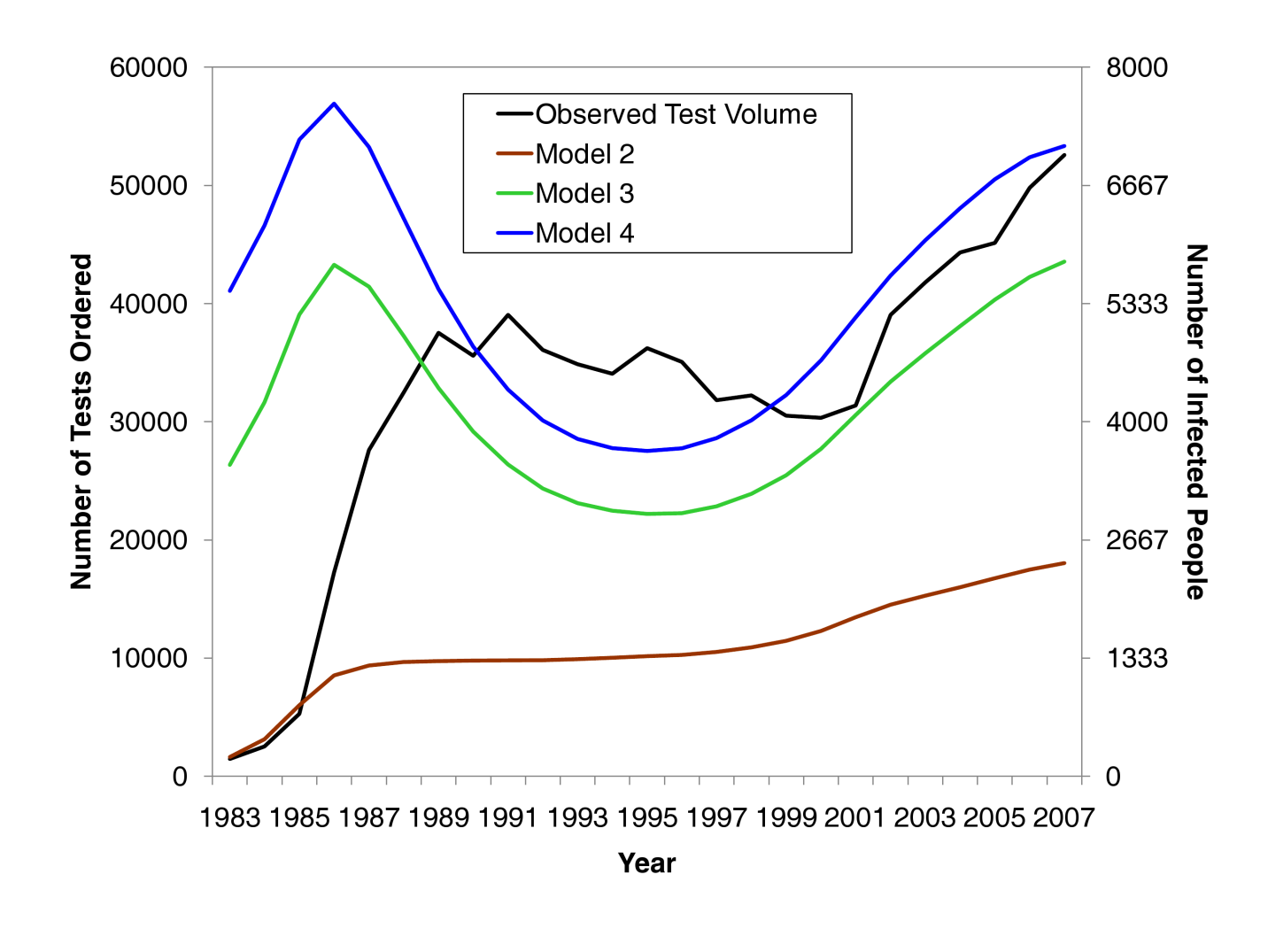
**
